# First Study of Monopisthocotylan Parasites of Cichlid Fishes in Togo: The Case of *Coptodon zillii* (Gervais, 1848), *Sarotherodon galilaeus* (Linnaeus, 1758) and *Oreochromis niloticus* (Linnaeus, 1758) From the Kara River Basin

**DOI:** 10.1155/japr/3448134

**Published:** 2026-11-15

**Authors:** Nazifou Maman, Enoutchy Fabrice Bouah, Amakoé Adjanke, Rodrigue Orobiyi Edéya Pèlèbè, Maarten P. M. Vanhove, Ibrahim Imorou Toko, Valentin N′douba

**Affiliations:** ^1^ Laboratory of Applied Agronomic and Biological Sciences (LaSABA) University of Kara Kara Togo; ^2^ Research Laboratory in Aquaculture and Aquatic Ecotoxicology (LaRAEAq) University of Parakou Parakou Benin univ-parakou.com; ^3^ Laboratory of Natural Environments and Biodiversity Conservation UFR Biosciences Félix Houphouët—Boigny University Abidjan Côte d’Ivoire; ^4^ Centre for Environmental Sciences Research Group Zoology: Biodiversity and Toxicology Hasselt University Diepenbeek Belgium uhasselt.be; ^5^ Freshwater Biology Operational Directorate Natural Environment Royal Belgian Institute of Natural Sciences Brussels Belgium naturalsciences.be

**Keywords:** *Cichlidogyrus*, Dactylogyridae, Monopisthocotyla, *Scutogyrus*

## Abstract

This study investigated the gill monopisthocotylan fauna of 150 cichlid specimens belonging to *Coptodon* Gervais, 1853, *Oreochromis* Günther, 1889 and *Sarotherodon* Rüppell, 1852, collected using a cast net from the Kara River. The parasitological examination revealed eight monopisthocotylan species belonging to Platyhelminthes, Monopisthocotyla, Dactylogyridae Bychowsky, 1937, distributed within *Cichlidogyrus* and *Scutogyrus* Pariselle and Euzet, 1995. The identified species were *Cichlidogyrus halli* Price and Kirk, 1967; *Cichlidogyrus dossoui* Douëllou, 1993; *Cichlidogyrus bouvii* Pariselle and Euzet, 1997; *Cichlidogyrus thurstonae* Ergens, 1981; *Cichlidogyrus tilapiae* Paperna, 1960; *Cichlidogyrus ergensi* Dossou, 1982; *Scutogyrus longicornis* Paperna and Thurston, 1969 and *Scutogyrus bailloni* Pariselle and Euzet, 1995. These findings represent the first record of monopisthocotylans infecting cichlids in Togo, more precisely in the Kara River basin, and contribute to a baseline for understanding the parasitic biodiversity of freshwater fishes. They also provide a reference for future monitoring of the health status of wild cichlid populations.

## 1. Introduction

Freshwater fisheries resources play a fundamental role in food security, nutrition and livelihoods across sub‐Saharan Africa [1]. Among these resources, cichlid fishes, particularly those belonging to *Coptodon* Gervais, 1853, *Oreochromis* Günther, 1889 and *Sarotherodon* Rüppell, 1852, rank among the most widely exploited species in artisanal fisheries and aquaculture systems in West Africa [2]. Their high economic value, rapid growth rates and remarkable ability to adapt to a wide range of environmental conditions make them species of choice for aquaculture development and sustainable fisheries management [1, 2]. Given their ecological and socio‐economic importance, freshwater fishes are exposed to various biological pressures, among which parasitic infections play a major role [3]. Monopisthocotylans, mostly ectoparasitic platyhelminths with a direct life cycle, are among the most common parasites of teleost fishes. They primarily colonise the gills, although they may also infest the skin and fins and, less frequently, other organs such as the nasal cavities, urinary bladder or stomach (e.g., members of *Enterogyrus* in cichlids) [4]. Their diversity, abundance and spatial distribution are closely linked to environmental conditions and host biological traits. Because these ectoparasites with a direct life cycle are permanently exposed to the surrounding water, their communities respond to changes in water quality and other environmental disturbances, which gives them strong potential as bioindicators of ecosystem health [5, 6]. From both biological and socio‐economic perspectives, monopisthocotylan infections may induce significant pathological effects in their hosts, including reduced growth, respiratory dysfunction and immune system impairment, particularly under heavy infestations [7, 8]. These effects may result in substantial economic losses in both natural fisheries and aquaculture settings [7, 9, 10]. Consequently, the study of these parasites is essential not only for understanding host–parasite interactions, but also for developing appropriate management strategies aimed at sustainably enhancing fish production. Across Africa, the diversity of monopisthocotylan parasites infecting cichlid fishes has been investigated in several major freshwater systems, although the intensity of research varies considerably among regions. In West Africa, numerous studies have been conducted in Côte d′Ivoire [11–14], Senegal [15–17] and Ghana [18]. In Central Africa, investigations have been reported from, among others, Cameroon [19, 20] and the Democratic Republic of Congo [21, 22]. In East Africa, studies from Kenya [23, 24] have further contributed to our understanding of monopisthocotylan diversity. The Lake Tanganyika basin has also received particular attention, with several studies revealing a substantial diversity of cichlid monopisthocotylans [25, 26]. *Cichlidogyrus*, in particular, represents a major component of the monopisthocotylan fauna associated with African cichlids.

In contrast, in Togo, the only available studies on monopisthocotylans, by Kritsky et al. [27, 28], are relatively old and concern noncichlid hosts (alestids and bagrid catfishes). To our knowledge, no study has yet been devoted to the monopisthocotylans parasitising cichlids in the Kara River basin.

In this context, the present study provides the first inventory of gill monopisthocotylans parasitising *Coptodon zillii* (Gervais, 1848), *Sarotherodon galilaeus* (Linnaeus, 1758) and *Oreochromis niloticus* (Linnaeus, 1758) in the Kara River basin. These three species are native to the Volta basin, to which the Kara River belongs [29, 30]; the parasites recorded here therefore represent new locality records within the native range of their hosts. This work thus contributes to filling a major gap in the knowledge of parasitic biodiversity in Togolese freshwater ecosystems.

## 2. Materials and Methods

### 2.1. Study Area

The Kara River is located in northern Togo, within the Kara Region (Figure 1). It is approximately 197 km long and drains a watershed of about 5,316 km^2^ within Togolese territory. The river is one of the main right‐bank tributaries of the Oti River, which represents the principal drainage system of northern Togo [29]. The Kara River rises in the Atacora Mountains in the Republic of Benin, flows through the city of Kara, then turns westward before joining the Oti River at the extreme western part of the region, near Koulifiékou, close to the Togo–Ghana border. Its channel is generally braided and characterised by numerous rocky outcrops, notably the Kara orthogneisses, which strongly influence the river′s morphology. The hydrological regime of the Kara River is irregular, with a major flood period occurring during the main rainy season, from March/April to mid‐June. Low‐water conditions prevail during the dry season, from November to March, reflecting the tropical Sudanian climate with contrasting seasons that characterise the region [29]. This marked seasonal variability strongly influences aquatic habitat dynamics and the spatial distribution of living organisms. For the present study, two sampling sites were selected along the Kara River based primarily on their accessibility. Site 1, located in the village of Wolou, lies on the Tchak′im bank (9°33 ^′^00.461 ^″^ N; 1°06 ^′^54.809 ^″^ E). Site 2, situated in the village of Sarakawa, is located on the Tèyo bank (9°34 ^′^58.068 ^″^ N; 1°03 ^′^20.004 ^″^ E). The three host species studied are native to the Volta basin, to which the Kara River belongs [29, 30].

**Figure 1:**
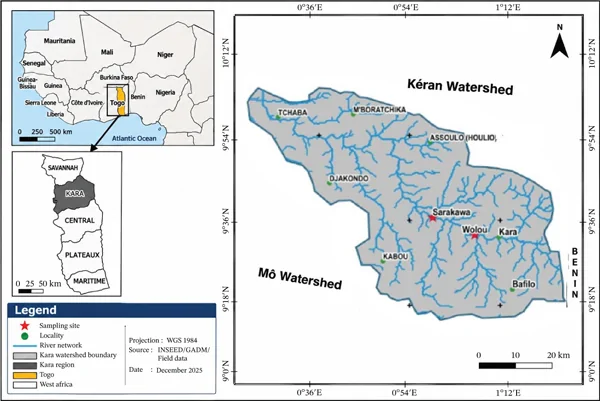
Location of the Kara River basin and the sampling sites.

### 2.2. Fish Sampling and Gill Collection

A total of 150 fish specimens were collected using a cast net during the sampling period from 1 to 6 August 2025. The fish were killed by keeping them cool in a cooler containing ice packs and identified to species level using the taxonomic keys of Paugy et al. [30]. Standard length was measured with an ichthyometer and body mass was recorded using an electronic balance. The gill apparatus of each fish was removed by making two incisions, one ventral and one dorsal and each gill arch was carefully excised. The gills were then labelled according to the classical protocol described by N′Douba [31], Bouah [4] and Kouadio [32]. The samples were stored in a cooler with ice and transported to the laboratory for parasitological examination.

### 2.3. Recovery and Identification of Monopisthocotylans

In the laboratory, after thawing, the gill arches were separated and placed in Petri dishes. Monopisthocotylans were detached from the gills by vigorous rinsing. Individual parasites were collected using fine entomological needles and directly mounted on microscope slides in a microdrop of ammonium picrate–glycerine mixture [33]. Each preparation was covered with a coverslip and, after complete diffusion of the mounting medium, sealed with Glyceel and labelled according to the corresponding gill arch. Observations and identifications were performed using a Motic BA310 light microscope equipped with an integrated camera. Monopisthocotylans were identified to species level using the taxonomic keys of Pariselle and Euzet [16] and by comparison with original species descriptions. Infection parameters (prevalence, mean intensity and mean abundance) follow the terminology of Bush et al. [34] (Table 1).

**Table 1:** Infection parameters of gill monopisthocotylans in *Coptodon zillii*, *Oreochromis niloticus* and *Sarotherodon galilaeus* from the Kara River.

Monopisthocotylan species	Host (*N*)	P (%) (95% CI)	MI	MA
*Cichlidogyrus ergensi*	Cz (10)	90.00 (55.4–99.5)	2.11	1.90
	Sg (120)	5.83 (2.8–11.6)	2.00	0.12
*Cichlidogyrus thurstonae*	On (20)	85.00 (62.8–95.8)	6.00	5.10
*Cichlidogyrus halli*	Sg (120)	91.67 (85.1–95.5)	10.25	9.40
*Cichlidogyrus tilapiae*	On (20)	15.00 (4.2–37.2)	3.00	0.45
	Sg (120)	2.50 (0.7–7.3)	1.00	0.03
*Cichlidogyrus bouvii*	Sg (120)	8.33 (4.5–14.9)	3.60	0.30
*Cichlidogyrus dossoui*	Sg (120)	75.00 (66.3–82.2)	3.52	2.64
*Scutogyrus longicornis*	On (20)	85.00 (62.8–95.8)	5.00	4.25
	Sg (120)	10.83 (6.1–17.8)	1.00	0.11
*Scutogyrus bailloni*	Sg (120)	28.33 (20.7–37.0)	1.91	0.54

Abbreviations: CI, 95% Sterne confidence interval (34, 35); Cz, *Coptodon zillii*; MA, mean abundance; MI, mean intensity; *N*, number of hosts examined; On, *Oreochromis niloticus*; P, prevalence; Sg, *Sarotherodon galilaeus*.

### 2.4. Statistical Analysis

For each monopisthocotylan species and host species, prevalence (P) (%) with 95% Sterne confidence intervals, mean intensity and mean abundance were calculated using QPweb [35]. Overall prevalence (proportion of fish infected by at least one monopisthocotylan species) was compared among host species using Fisher′s exact test. As parasite counts were recorded as totals per parasite species and host species rather than per individual fish, intensity ranges, confidence intervals for mean intensity and mean abundance and species accumulation curves could not be computed.

## 3. Results

Among the 150 cichlid specimens examined, infection levels varied among host species. Out of the 10 *C*. *zillii* examined, nine individuals (90%) were infected, harbouring a total of 19 monopisthocotylans, corresponding to a mean intensity of 2.11 parasites per infected fish. In *O*. *niloticus*, 17 of the 20 specimens examined (85%) were parasitised, with a total of 196 monopisthocotylans, giving a mean intensity of 11.53 parasites per infected fish. The highest infection level was recorded in *S*. *galilaeus*, in which 110 out of 120 individuals (91.7%) were infected. A total of 1576 gill monopisthocotylans were recovered from this host, corresponding to a mean intensity of 14.33 parasites per infected fish. Overall prevalence did not differ significantly among the three host species (Fisher′s exact test, *p* = 0.76). Morpho‐anatomical examination of the monopisthocotylans collected from the gills of the three cichlid species revealed a total of eight gill monopisthocotylan species. These parasites belong to the order Dactylogyridea, family Dactylogyridae and to the genera *Cichlidogyrus* Paperna, 1960 and *Scutogyrus* Pariselle and Euzet, 1995 (Table 1).

The monopisthocotylans recorded were primarily distinguished by the morphology of their haptors and copulatory organs (Figures 2, 3 and 4). In *C*. *zillii*, only one species was recovered, namely *Cichlidogyrus ergensi* Dossou, 1982. In *O*. *niloticus*, three species were identified: *Cichlidogyrus thurstonae* Ergens, 1981, *Cichlidogyrus tilapiae* Paperna, 1960 and *Scutogyrus longicornis* Paperna and Thurston, 1969. In contrast, *S*. *galilaeus* exhibited the most species‐rich parasite community, with seven species distributed across two genera: *Cichlidogyrus* (*Cichlidogyrus halli* Price and Kirk, 1967; *Cichlidogyrus bouvii* Pariselle and Euzet, 1997; *Cichlidogyrus dossoui* Douëllou, 1993; *Cichlidogyrus tilapiae* Paperna, 1960 and *C*. *ergensi* Dossou, 1982) and *Scutogyrus* (*Scutogyrus bailloni* Pariselle and Euzet, 1995 and *S*. *longicornis* Paperna and Thurston, 1969). Notably, *C*. *ergensi* was shared by *C*. *zillii* and *S*. *galilaeus*, whereas *C*. *tilapiae* and *S*. *longicornis* were common to *S*. *galilaeus* and *O*. *niloticus*.

**Figure 2:**
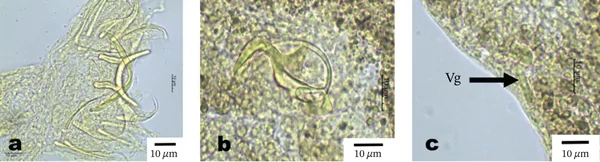
Micrographs of the sclerotised structures of the haptor and copulatory organs of monopisthocotylans collected from *Coptodon zillii* in the Kara River: (a) haptor of *Cichlidogyrus ergensi*, (b) male copulatory organ of *C*. *ergensi* and (c) vagina (Vg) of *C*. *ergensi*.

**Figure 3:**
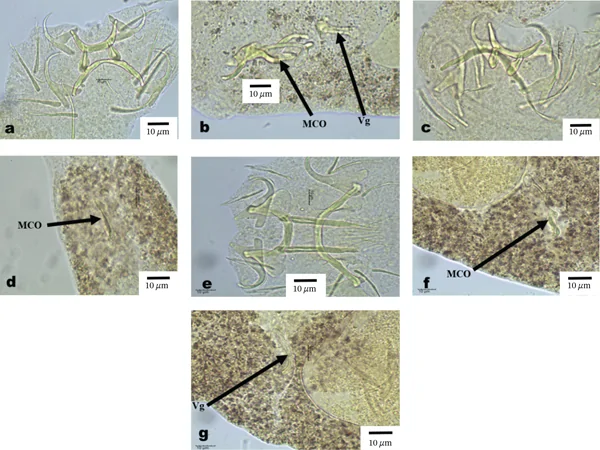
Micrographs of the sclerotised structures of the haptor and copulatory organs of monopisthocotylans collected from *Oreochromis niloticus* in the Kara River: (a) haptor of *Cichlidogyrus thurstonae*, (b) male copulatory organ (MCO) and vagina (Vg) of *C*. *thurstonae*, (c) haptor of *Cichlidogyrus tilapiae*, (d) MCO of *C*. *tilapiae*, (e) haptor of *Scutogyrus longicornis*, (f) MCO of *S*. *longicornis* and (g) vagina of *S*. *longicornis*.

**Figure 4:**
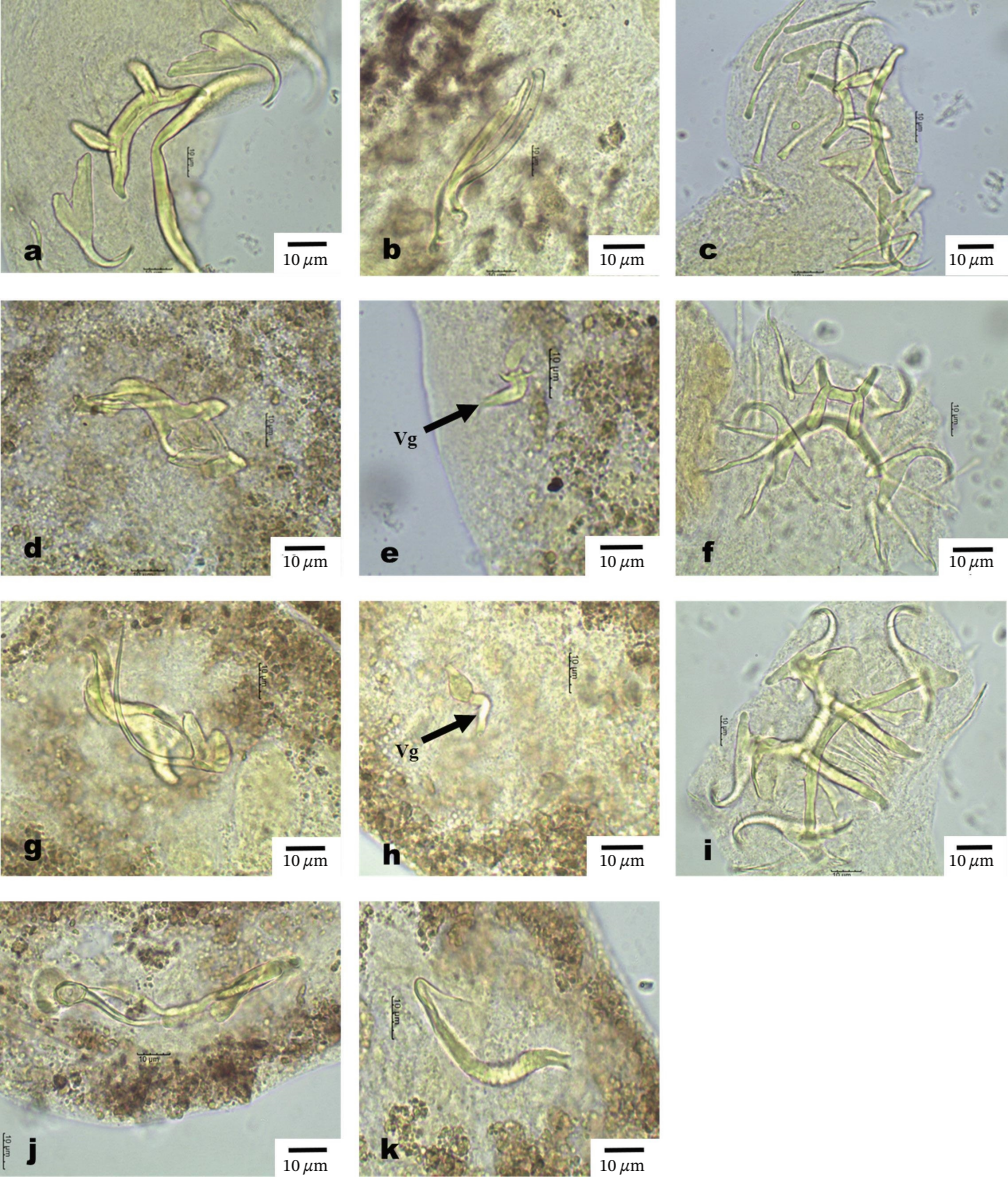
Micrographs of the sclerotised structures of the haptor and copulatory organs of monopisthocotylans collected from *Sarotherodon galilaeus* in the Kara River: (a) haptor of *Cichlidogyrus halli*, (b) male copulatory organ of *C*. *halli*, (c) haptor of *Cichlidogyrus dossoui*, (d) male copulatory organ of *C*. *dossoui*, (e) vagina (Vg) of *C*. *dossoui*, (f) haptor of *Cichlidogyrus bouvii*, (g) male copulatory organ of *C*. *bouvii*, (h) vagina of *C*. *bouvii*, (i) haptor of *Scutogyrus bailloni*, (j) male copulatory organ of *S*. *bailloni* and (k) vagina of *S*. *bailloni*.

## 4. Discussion

The present study is the first contribution to the knowledge of monopisthocotylan communities associated with cichlids in Togo, specifically in the Kara River basin. It revealed a species richness of one, three and seven monopisthocotylan species of *Cichlidogyrus* and *Scutogyrus* infecting the gills of *C*. *zillii*, *O*. *niloticus* and *S*. *galilaeus*, respectively. The differences in parasite richness among host species likely result from the combined effects of methodological and biological factors. On the one hand, sampling effort directly influences estimates of parasite diversity [36], making the uneven sample sizes in the present study relevant (10 *C*. *zillii*, 20 *O*. *niloticus* and 120 *S*. *galilaeus* examined). On the other hand, host biological and behavioural traits, such as body size, habitat use, social behaviour and life‐history strategies, strongly condition opportunities for parasite transmission [37]. In this study, the contrasting infection patterns observed among *C*. *zillii*, *O*. *niloticus* and *S*. *galilaeus* may also partly reflect their distinct ecological traits, although these traits were not analysed in the present study. In *C*. *zillii*, only *C*. *ergensi* was recorded, whereas three to five monopisthocotylan species have been reported from this host in Côte d′Ivoire [12–14]. This difference most likely reflects the small number of *C*. *zillii* examined (*n* = 10) [36], all hosts having been examined using the same protocol.

The phylogenetic relationships between cichlids and their monopisthocotylans may also play a role in shaping parasitic communities. Cophylogenetic studies on *Cichlidogyrus* and *Scutogyrus* indeed demonstrate the importance of evolutionary interactions between parasites and their hosts in determining host specificity and the composition of parasitic assemblages, although this relationship is not strictly phylogenetically determined [26, 38, 39]. However, the sharing of *C*. *ergensi*, *C*. *tilapiae* and *S*. *longicornis* among hosts is more likely explained by host switching, which is frequent in generalist species such as *C*. *tilapiae* and *C*. *halli* [39, 40], and by the co‐occurrence of the hosts in the same habitat. In *C*. *zillii*, the single species recorded in the Kara River contrasts with the three to five species reported from this host in Côte d′Ivoire by Blahoua et al. [12, 13] (Lake Ayamé I and Lobo River) and Adou et al. [14] (Lake Ayamé II). Besides the small sample size, these differences may reflect the sampling season (a single sampling event in August), host population density and local ecological conditions.

Local ecological conditions such as water quality, habitat stability, habitat structure and host availability, which were not measured in the present study, are known to play a crucial role in monopisthocotylan survival, development and transmission. Thus, for a given host species, parasite community composition and richness may vary considerably between ecosystems, emphasising the importance of environmental context in host–parasite dynamics [41]. For instance, Ibrahim [42] recorded eight monopisthocotylan species on the gills of *C*. *zillii*, whereas Lim et al. [43] reported six species on *O*. *niloticus* in Malaysia.

The high parasite richness observed in *S*. *galilaeus* likely reflects its ecological plasticity and its central role within the trophic network of the studied ecosystem. In West Africa, several studies have shown that *S*. *galilaeus*, due to its wide geographical distribution, high abundance and frequent interactions with other cichlids, is particularly exposed to a diverse parasite fauna [44, 45]. These characteristics increase the probability of contact with infective stages and favour the establishment of complex parasite assemblages. The coexistence of multiple species of *Cichlidogyrus* and *Scutogyrus* on the same host suggests functional and ecological diversification of monopisthocotylans. This differentiation could potentially reduce interspecific competition by promoting the exploitation of distinct micro‐niches. Studies have indeed shown a non‐random spatial distribution of several species of monopisthocotylans on the gill arches and suggest a microhabitat preference in certain species of *Cichlidogyrus* [11, 13, 46]. The distribution of shared parasite species among hosts highlights a relative host specificity. *C*. *ergensi* was common to *C*. *zillii* and *S*. *galilaeus*, whereas *C*. *tilapiae* and *S*. *longicornis* were shared by *S*. *galilaeus* and *O*. *niloticus*. This overlap suggests that some monopisthocotylans can infect several phylogenetically related hosts, particularly when they co‐occur in the same environment. Similar polyparasitism has been reported in *S*. *galilaeus* from the Loumbila reservoir near Ouagadougou, Burkina Faso [47]. In wild populations, where parasite densities are generally low, competition for attachment sites is limited, which may facilitate the coexistence of several monopisthocotylan species on the same host [48, 49].

From an epidemiological perspective, the profile observed in this study (high prevalence but low intensity) reflects a dynamic equilibrium between host and parasite [7]. However, certain species, such as *C*. *halli* and *C*. *dossoui*, which are very common in *S. galilaeus*, could become pathogenic under stressful conditions (overpopulation, pollution and temperature fluctuations), as has been demonstrated in other farmed cichlids [9, 17]. In addition, transfers of *O*. *niloticus* between fish farms and natural water bodies may favour the introduction of non‐native parasites or parasite lineages; the present data provide a baseline against which such introductions could be detected. Finally, the present study was restricted to gill monopisthocotylans; other ectoparasites and endohelminths of these cichlids, which require different examination protocols, should be investigated in future studies.

## 5. Conclusion

The present study recorded eight gill monopisthocotylan species on three native cichlids of the Kara River basin, with species richness differing among host species (one on *C*. *zillii*, three on *O*. *niloticus* and seven on *S*. *galilaeus*), potentially partly as a result of unequal sample sizes. This work provides the first baseline on the monopisthocotylan communities of cichlids in Togo and fills a knowledge gap regarding parasitic biodiversity in Togolese freshwater ecosystems. It may underpin future monitoring of the health status of wild and farmed cichlid populations in the Kara River basin.

## Funding

No funding was received for this manuscript.

## Conflicts of Interest

The authors declare no conflicts of interest.

## Data Availability

The data supporting the findings of this study are available from the corresponding author upon request.
